# Training in Use of Brain–Machine Interface-Controlled Robotic Hand Improves Accuracy Decoding Two Types of Hand Movements

**DOI:** 10.3389/fnins.2018.00478

**Published:** 2018-07-11

**Authors:** Ryohei Fukuma, Takufumi Yanagisawa, Hiroshi Yokoi, Masayuki Hirata, Toshiki Yoshimine, Youichi Saitoh, Yukiyasu Kamitani, Haruhiko Kishima

**Affiliations:** ^1^Department of Neurosurgery, Graduate School of Medicine, Osaka University, Suita, Japan; ^2^Department of Neuroinformatics, ATR Computational Neuroscience Laboratories, Seika-cho, Japan; ^3^Center for Information and Neural Networks, National Institute of Information and Communications Technology, Suita, Japan; ^4^Institute for Advanced Co-Creation Studies, Osaka University, Suita, Japan; ^5^Endowed Research Department of Clinical Neuroengineering, Global Center for Medical Engineering and Informatics, Osaka University, Suita, Japan; ^6^Department of Mechanical Engineering and Intelligent Systems, University of Electro-Communications, Chofu, Japan; ^7^Department of Neuromodulation and Neurosurgery, Graduate School of Medicine, Osaka University, Suita, Japan; ^8^Graduate School of Informatics, Kyoto University, Kyoto, Japan

**Keywords:** brain-machine interface, robotic hand, magnetoencephalography, cortical plasticity, neurofeedback, closed-loop training, online decoding

## Abstract

**Objective:** Brain-machine interfaces (BMIs) are useful for inducing plastic changes in cortical representation. A BMI first decodes hand movements using cortical signals and then converts the decoded information into movements of a robotic hand. By using the BMI robotic hand, the cortical representation decoded by the BMI is modulated to improve decoding accuracy. We developed a BMI based on real-time magnetoencephalography (MEG) signals to control a robotic hand using decoded hand movements. Subjects were trained to use the BMI robotic hand freely for 10 min to evaluate plastic changes in the cortical representation due to the training.

**Method:** We trained nine young healthy subjects with normal motor function. In open-loop conditions, they were instructed to grasp or open their right hands during MEG recording. Time-averaged MEG signals were then used to train a real decoder to control the robotic arm in real time. Then, subjects were instructed to control the BMI-controlled robotic hand by moving their right hands for 10 min while watching the robot's movement. During this closed-loop session, subjects tried to improve their ability to control the robot. Finally, subjects performed the same offline task to compare cortical activities related to the hand movements. As a control, we used a random decoder trained by the MEG signals with shuffled movement labels. We performed the same experiments with the random decoder as a crossover trial. To evaluate the cortical representation, cortical currents were estimated using a source localization technique. Hand movements were also decoded by a support vector machine using the MEG signals during the offline task. The classification accuracy of the movements was compared among offline tasks.

**Results:** During the BMI training with the real decoder, the subjects succeeded in improving their accuracy in controlling the BMI robotic hand with correct rates of 0.28 ± 0.13 to 0.50 ± 0.11 (*p* = 0.017, *n* = 8, paired Student's *t*-test). Moreover, the classification accuracy of hand movements during the offline task was significantly increased after BMI training with the real decoder from 62.7 ± 6.5 to 70.0 ± 11.1% (*p* = 0.022, *n* = 8, *t*_(7)_ = 2.93, paired Student's *t-*test), whereas accuracy did not significantly change after BMI training with the random decoder from 63.0 ± 8.8 to 66.4 ± 9.0% (*p* = 0.225, *n* = 8, *t*_(7)_ = 1.33).

**Conclusion:** BMI training is a useful tool to train the cortical activity necessary for BMI control and to induce some plastic changes in the activity.

## Introduction

Brain–machine interfaces (BMIs) can reconstruct motor function in paralyzed subjects (Hochberg et al., 2006, 2012; Yanagisawa et al., 2012a; Collinger et al., 2013; Bouton et al., 2016) as well as induce functional alterations in cortical activity (Ganguly et al., 2011; Wander et al., 2013; Orsborn et al., 2014; Yanagisawa et al., 2016). A BMI works by first recording neural activity and then converting the recorded activity into control of some machine, such as a robotic hand or computer (Yanagisawa et al., 2009, 2011, 2012a,b; Nakanishi et al., 2013, 2014; Fukuma et al., 2015, 2016). Recent studies demonstrated that neurofeedback training using BMI induces plastic changes in neural activities in accordance with some functional alterations in the neural system. The neurofeedback of decoded information using functional magnetic resonance imaging (fMRI) demonstrated that the training induced alteration of cortical activities in accordance with alterations in cognition (Shibata et al., 2011, 2016; Amano et al., 2016; Ordikhani-Seyedlar et al., 2016). In addition, using a certain power spectrum of electroencephalographic signals, motor rehabilitation was improved in stroke patients (Shindo et al., 2011; Ramos-Murguialday et al., 2013). Moreover, we recently reported that BMI training to control a robotic hand induced plastic changes in the motor cortical representation of phantom limb pain patients and changed their pain in accordance with the plastic changes (Yanagisawa et al., 2016).

Such plastic changes are attributed to reinforcement learning with the BMI feedback (Watanabe et al., 2017). The closed-loop system with decoded information enables subjects to modulate the decoded information based on the feedback as a reward. Therefore, we expect that training to use a BMI based on the decoding information would improve the decoding accuracy better than training to use a BMI that is not based on the decoding information.

In this study, we demonstrate that BMIs based on magnetoencephalography (MEG) signals precisely decode hand movements in real time (Bradberry et al., 2009; Toda et al., 2011; Fukuma et al., 2015) and training to use the BMIs induces plastic changes in cortical activity of healthy subjects (Nishimura et al., 2013; Clancy et al., 2014; Luu et al., 2017), especially in the accuracy to decode hand movements.

## Subjects and methods

### Subjects

Nine young right-handed volunteers with normal neurological function (2 males and 7 females; mean age, 24.1 years; range, 21–30 years) participated in this study. The study adhered to the Declaration of Helsinki and was performed in accordance with protocols approved by the Ethics Committee of Osaka University Clinical Trial Center (no. 12107, UMIN000010180). All participants were informed of the purpose and possible consequences of this study, and written informed consent was obtained. We recruited subjects aged 20 years and older with normal neurological functioning. Inclusion criteria did not consider gender, race or any special experience.

### MEG recording

**Figure 1:**
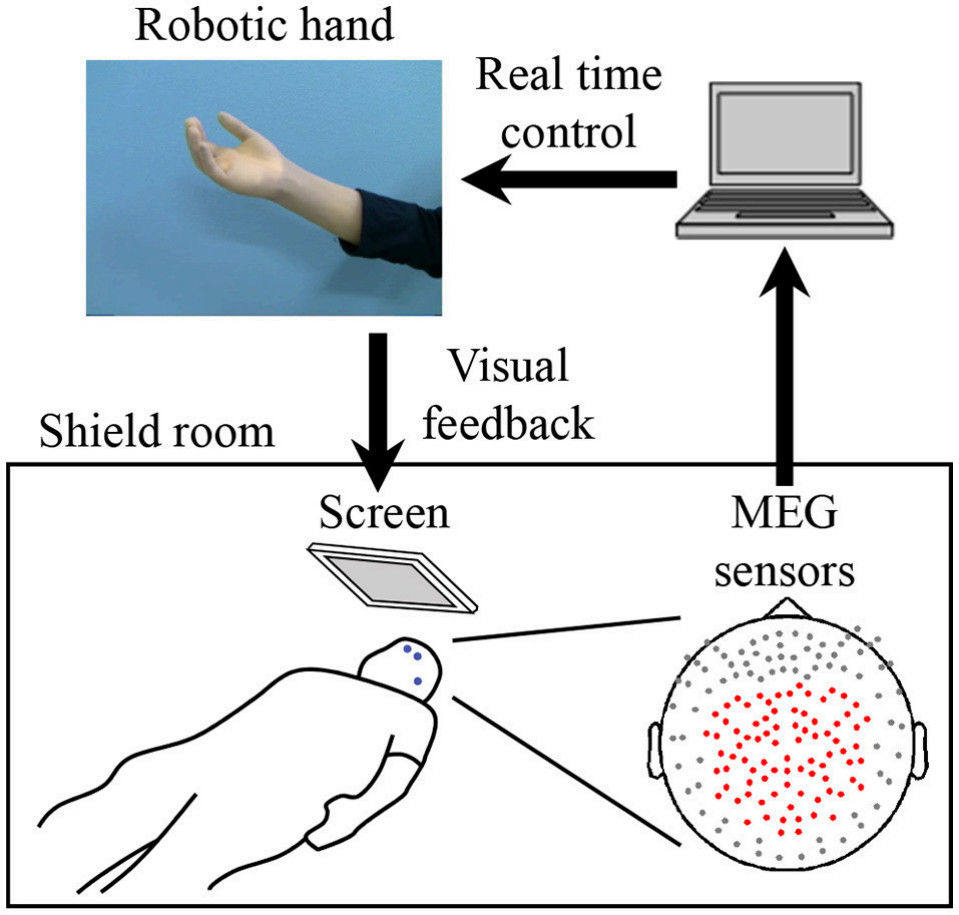
System overview for training to use a robotic hand. MEG signals from 84 parietal sensors (shown with red dots) were acquired in real-time to decode performed movement. The robotic hand was controlled according to the results of the decoder. The participant received visual feedback of the robotic hand presented on the screen. Blue dots on the participant's face denote head marker coils used to determine position and orientation of MEG sensors relative to the head. Three marker coils (at the center of the forehead, above the left eyebrow, and on the left preauricular area) are shown.

For the MEG recording, subjects were in the supine position with the head centered in the gantry. A projection screen in front of the face provided stimuli using a visual stimulus presentation system (Presentation; Neurobehavioral Systems, Albany, CA, USA) and a liquid crystal projector (LVP-HC6800; Mitsubishi Electric, Tokyo, Japan) (Figure 1). MEG signals were measured by a 160-channel whole-head MEG equipped with coaxial-type gradiometers (MEGvision NEO; Yokogawa Electric Corporation, Kanazawa, Japan) housed in a magnetically shielded room.

The MEG signals were sampled at 1,000 Hz with an online low-pass filter at 200 Hz and acquired online by FPGA DAQ boards (PXI-7854R; National Instruments, Austin, TX, USA) after passing through an optical isolation circuit. For the online control of the robotic hand, signals from 84 selected sensors (Figure 1) were used, except for one experiment in which 81 sensors were used for technical reasons. The same 84 sensors were used for offline analysis. Subjects were instructed to not move the head to avoid motion artifacts. A cushion was placed under the elbows to reduce motion artifacts.

Five head marker coils were attached to the subject's face before beginning the MEG recording, to provide the position and orientation of MEG sensors relative to the head (Figure 1). The positions of the five marker coils were measured to evaluate differences in the head position before and after each MEG recording. The maximum acceptable difference was 5 mm.

We also recorded electromyograms of the face and forearm to monitor muscle activities. Subjects were monitored by two video cameras to confirm their arousal.

### Experimental design

A crossover trial consisting of two experiments was performed with a washout period of more than 2 weeks. Each experiment consisted of three tasks, an offline task (pre-BMI), BMI training, and an offline task (post-BMI). For each training task, the participant controlled the robotic hand using two different decoders: a real decoder and a sham decoder. To balance which decoder type was selected first, the order for the real and sham decoders was randomized. Subjects were not informed about the order. Seven subjects participated in both experiments, one subject only participated in the experiment with the real decoder, and another only participated in the experiment with the sham decoder.

First, in the pre-BMI offline task, the subjects attempted to move their right hands (grasping and opening) at the presented times (Yanagisawa et al., 2012a) while MEG signals of the selected sensors were recorded (Figure 1). The subjects were visually instructed which movement to perform with the Japanese word for “grasp” or “open.” After the instruction for movement type, four execution cues were given to the subject every 5.5 s. The execution cue was given both visually and aurally, and was presented 40 times for each movement type. The order of the requested movement type was randomized. We instructed the subjects to slightly move the hand once at the cued time, without moving other body parts.

The MEG signals from the selected sensors were recorded during the task (Figure 1) and then time-averaged using windows of 500 ms from −2,000 to 1,000 ms at 100-ms intervals, with respect to the time of the execution cues. The averaged signals were converted into *z*-scores using the mean and standard deviations estimated from the initial 50 s of the offline task. The acquired *z*-scores were used to construct the decoder to control the robotic hand (Fukuma et al., 2015).

During the BMI training task, the subjects were instructed to control the prosthetic hand in real time using the trained decoder. The screen fixed in front of the subject showed a picture of the robotic hand in real time as visual feedback (Figure 1). Subjects were instructed to control the robotic hand freely for 10 min to improve their ability to control it by moving their hands (see Supplementary Video 1). Just before starting the training, the experimenter changed the threshold for detecting movement onset, because the threshold estimated from the offline task was sometimes too low, resulting in the detection of movement onsets even during the resting state in the online task. The other parameters estimated from the offline task were not changed (Fukuma et al., 2016). The selected parameters were fixed for the 10 min of training. The post-BMI offline task was performed in the same way as the pre-BMI task, after the BMI training task.

The BMI training to control the robotic hand was performed as a randomized crossover trial consisting of two training sessions on different days. Each training session was performed with two different decoders to control the robotic hand: a real decoder and a sham decoder. Using the *z-*scored MEG sensor signals of the offline tasks to move the right hand, we constructed a decoder to infer hand movements at an arbitrary time, in order to control the robotic hand in real time (Fukuma et al., 2015). Each experiment was performed after more than 2 weeks had passed since the previous experiment. For the experiments with the real decoder and sham decoder, the order of the experiments was randomly assigned to the subjects. The experimenter was not blinded to the group allocation.

At the time of enrollment in this trial, we instructed the subjects to use their brain activity to control the robotic hand, but they were not informed of the decoder they used.

### Decoder to control the prosthetic hand

MATLAB R2013a (Mathworks, Natick, MA, USA) was used to calculate the decoding parameters and for online robotic hand control. First, MEG signals from the 84 selected sensors during the offline task were averaged in a 500-ms time window and converted to the *z*-score using the mean and standard deviations estimated from the initial 50 s of data during the offline task. The time-averaged MEG signals were calculated for the period from −2,000 to 1,000 ms at 100-ms intervals according to the execution cue.

The *z-*scored signals from the offline task were used to train the online decoder, which consisted of an onset detector and class decoder, to control the robotic hand online in the following BMI training task. The class decoder was trained at the peak classification accuracy of the offline task by the support vector machine (SVM). The onset detector was trained using the *z-*scored signals to differentiate time period of the hand movement from period of resting by SVM and Gaussian process regression (GPR). The details of the construction of the decoder are available in our previous reports (Fukuma et al., 2015; Yanagisawa et al., 2016).

Here, we constructed two types of online decoders depending on the data used to train the decoder. The real decoder was trained by the MEG signals of the offline task to move the hand. The sham decoder was trained by the MEG signals of the same offline task with randomized types of movements (grasp or open).

### Evaluation of online BMI control

The movements of subject's hand and robotic hand were evaluated from the video recording. We counted the subject's hand movements. Then, we evaluated the robotic hand movements within 1 s after each movement of the subject's hand. If the robotic hand moved into the same posture (grasp or open) as the subject's hand, we counted the movement as correctly controlled movement. The correct rate of BMI control was evaluated by the number of correctly controlled movements divided by the total number of hand movements. The correct rate was counted for 1 min at the beginning and at the end of the 10-min training.

### Cortical current estimation by VBMEG

A polygonal model of the cortical surface was constructed based on structural MRI (T1-weighted; Signa HDxt Excite 3.0T; GE Healthcare UK Ltd., Buckinghamshire, UK) using the Freesurfer software (Martinos Center Software) (Dale et al., 1999). To align MEG data with individual MRI data, we scanned the three-dimensional facial surface and 50 points on the scalp of each participant (FastSCAN Cobra; Polhemus, Colchester, VT, USA). Three-dimensional facial surface data were superimposed on the anatomical facial surface provided by the MRI data. The positions of five marker coils before each recording were used to estimate cortical current with variational Bayesian multimodal encephalography (VBMEG) (Sato et al., 2004). VBMEG is free software for estimating cortical currents from MEG data (ATR Neural Information Analysis Laboratories, Kyoto, Japan; Cohen et al., 1991; Yoshioka et al., 2008). VBMEG estimated 4004 single current dipoles that were equidistantly distributed on and perpendicular to the cortical surface. An inverse filter was calculated to estimate the cortical current of each dipole from the selected 84 MEG sensor signals. The hyperparameters m0 and γ0 were set to 100 and 10, respectively. The inverse filter was estimated by using MEG signals in all trials from 0 to 1000 ms in the offline task, with the baseline of the current variance estimated from the signals from −1,500 to −500 ms. The filter was then applied to sensor signals in each trial to calculate cortical currents.

### Evaluation of cortical representation

We evaluated the cortical representation during the offline task using cortical current source estimation. First, VBMEG was used to estimate the cortical currents from the obtained MEG signals. Next, the estimated cortical currents were averaged using a 500-ms window starting from the execution cue and compared between two types of movements with a one-way analysis of variance (ANOVA) for each vertex. The *F*-value of the ANOVA was averaged for all subjects and color-coded on the normalized brain surface.

### Evaluation of classification accuracy of movement types in the offline task

A nested cross-validation (Quian Quiroga and Panzeri, 2009) was performed with a linear support vector machine using the *z*-scores of the MEG signals from selected sensors (Fukuma et al., 2015) to evaluate the accuracy of classifying the performed movement types. The *z*-scores from 11 time windows (ranging from −500 to 500 ms at 100-ms intervals, with respect to the timing of the instruction to move) were concatenated to form a decoding feature. To calculate the classification accuracy, 10-fold cross-validation was applied. For each fold, the testing data set was classified with a decoder that was trained completely independently from the testing data set. To optimize hyperparameters of the decoder independently from the testing data set, another 10-fold cross-validation was applied to the training data set so that hyperparameters that achieved the highest classification accuracy within the training data set were selected. Finally, classification accuracies during the two offline tasks before and after the BMI training session were compared using a paired Student's *t*-test. Significance threshold for this *t*-test was set to 0.025, because this study employs two *t*-tests: one for training with a real decoder and another with a sham decoder (Bonferroni correction).

## Results

### BMI training with a robotic hand

**Figure 2:**
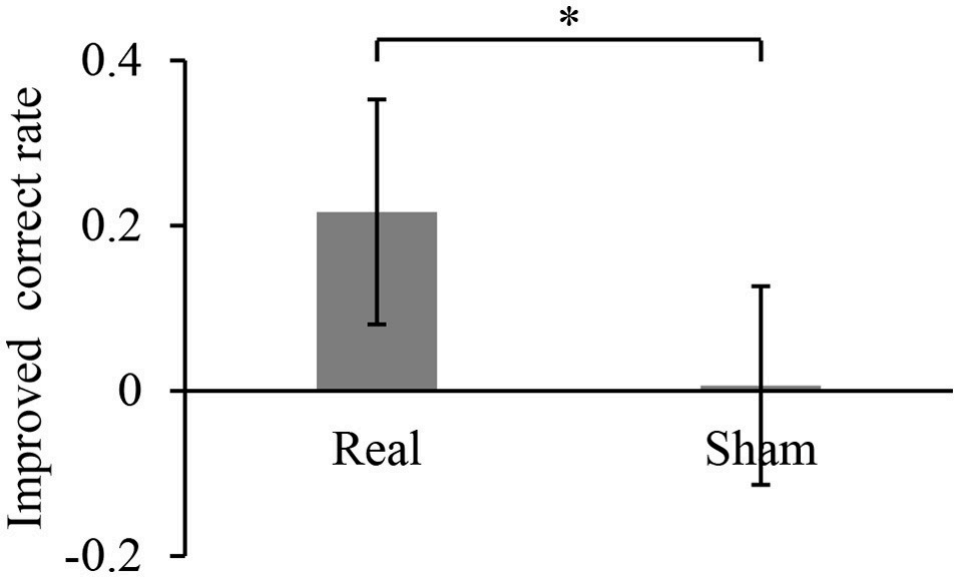
Improved accuracy of controlling the robotic hand during online BMI training. The correct rate for robotic hand control was calculated for the first 1 min of the training and the last 1 min of the 10-min training. Each bar shows the averaged improvement of the correct rate for the training with real and sham decoder. Error bars are 95% confidence intervals of the improved correct rate. ^*^p < 0.05 significant difference between two different decoders (unpaired Student's *t*-test).

During the 10-min BMI training, the accuracy in controlling the robotic hand was improved. The hand movements at an arbitrary timing were successfully detected and classified, with a correct rate of 0.28 ± 0.13 during the first 1 min of the BMI training with the real decoder. The correct rate increased significantly to 0.50 ± 0.11 for the final 1 min of the BMI training (*p* = 0.017, *n* = 8, paired Student's *t*-test). On the other hand, the correct rates during the BMI training with the random decoder were not significantly changed among the first 1 min and the last 1 min (0.51 ± 0.13 to 0.52 ± 0.10, *p* = 0.92, *n* = 8, paired Student's *t*-test). Notably, the increase of the correct rate during the BMI training with the real decoder was significantly larger than that during the BMI training with the random decoder (Figure 2). Also, it should be noted that correct rates during the first 1 min of the BMI training were not significantly different between the BMI trainings with the real decoder and the random decoder (*p* = 0.11, *n* = 7, paired Student's *t*-test).

### BMI training changed the cortical representation of hand movements

**Figure 3:**
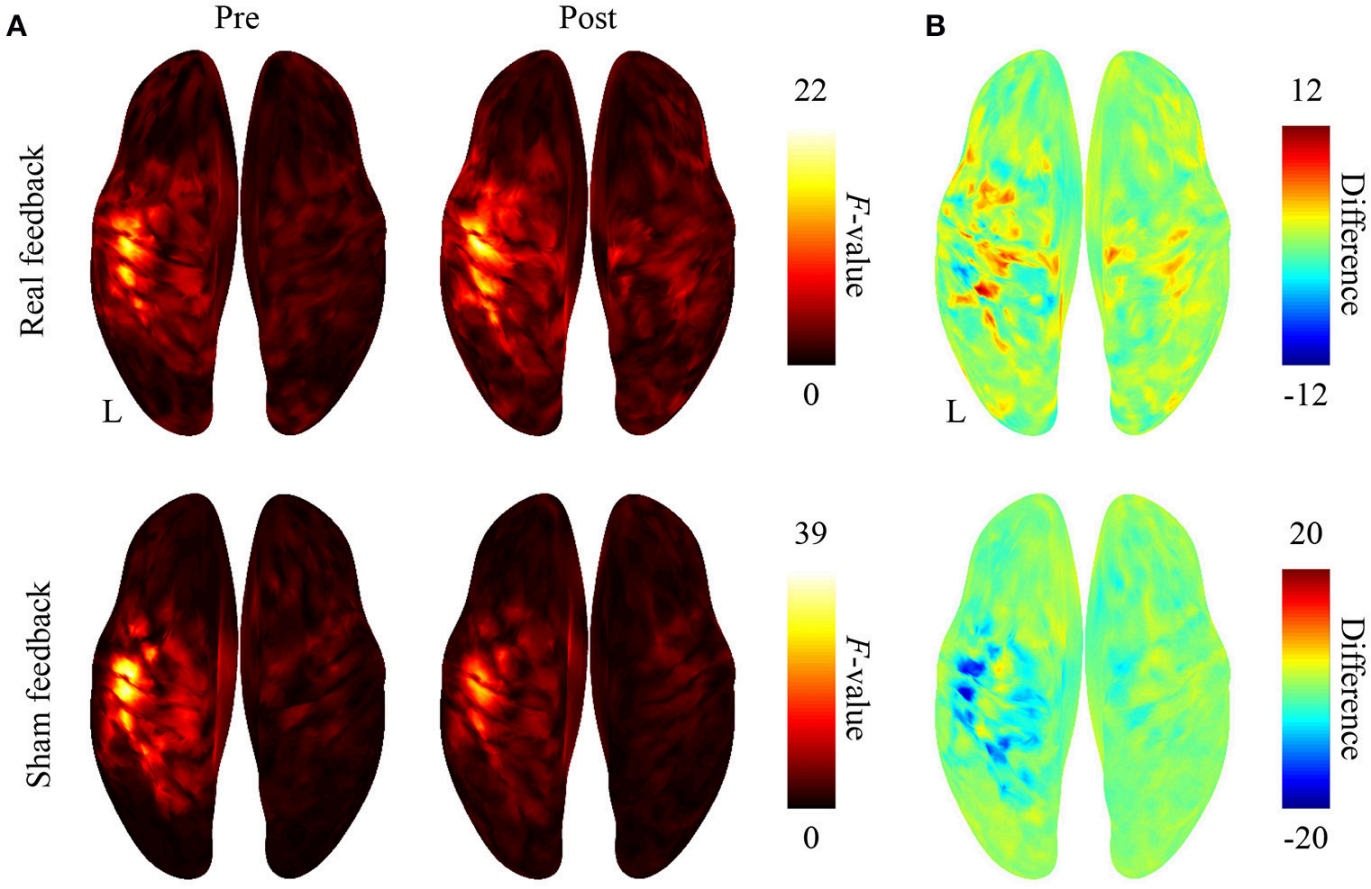
Difference in cortical activation evoked by two types of movements during the offline task. **(A)** The averaged *F*-values of one-way ANOVA between 500-ms time-averaged cortical currents estimated during hand grasping or opening were color-coded and plotted on the normalized brain surface. **(B)** The differences of *F*-values shown in plot **(A)** were color-coded on the normalized brain surface.

After BMI training with the real decoder, the *F*-values increased in the contralateral sensorimotor cortex (Figure 3A), although the difference of the *F*-values (Figure 3B) between pre-BMI and post-BMI offline tasks was not statistically significant (*p* > 0.05, paired *t*-test, FDR corrected). After training with the random decoder, the *F*-value of the contralateral sensorimotor cortex did not increase (Figures 3A,B), although the subject was instructed in the same way as during the experiment with real decoder. These findings suggest that the BMI training with the real decoder increased the discriminability of the cortical activity representing the hand movements.

### BMI training altered classification accuracy of hand movements

**Figure 4:**
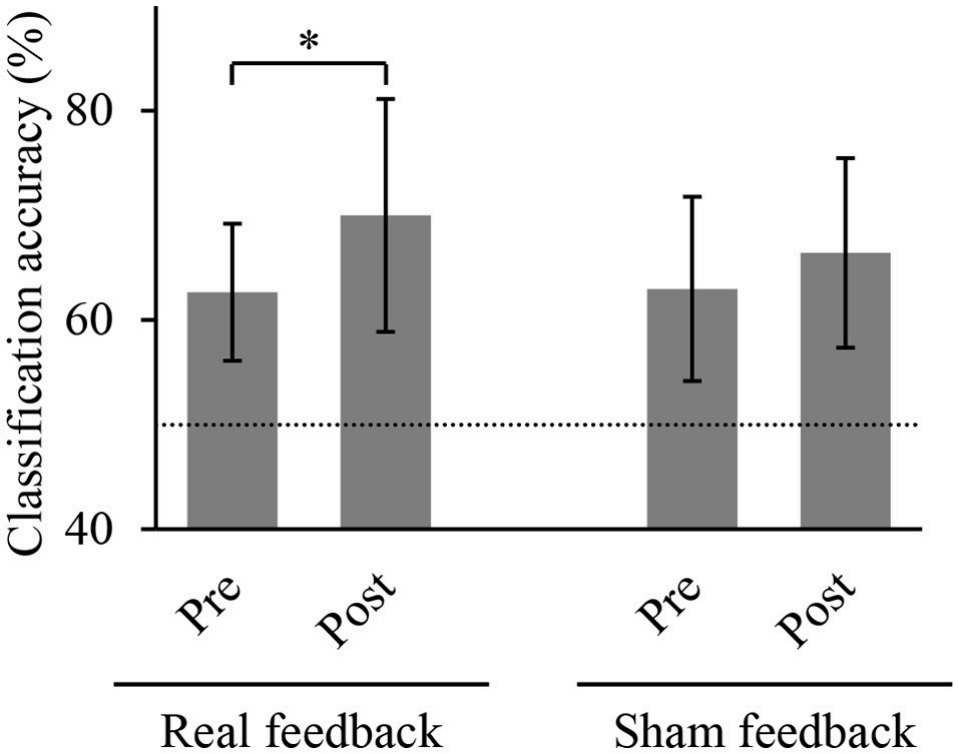
Classification accuracy of hand movements before and after training. Each bar shows the averaged classification accuracy of hand movements during the offline task. Error bars are 95% confidence intervals of classification accuracy. Dotted line denotes chance level. ^*^p < 0.05 significant difference between offline tasks before and after 10-min BMI training with feedback (paired Student's *t*-test with Bonferroni correction).

We compared the accuracies for classifying the hand movements using the *z*-scored MEG signals at the selected sensors. Figure 4 shows the classification accuracies of hand movements in the offline task before and after training task. The accuracy significantly increased after BMI training with the real decoder from 62.7 ± 6.5 to 70.0 ± 11.1% (*p* = 0.022, *n* = 8, *t*_(7)_ = 2.93, paired Student's *t-*test). In contrast, the BMI training with the random decoder did not increase the accuracy from 63.0 ± 8.8 to 66.4 ± 9.0% (*p* = 0.225, *n* = 8, *t*_(7)_ = 1.33). The BMI training with the real decoder significantly improved the cortical activity to decode the hand movements.

## Discussion

Our findings demonstrated that MEG-based BMI training to control a robotic hand significantly improved the accuracy to control the robotic hand and induced significant changes of the cortical representation of hand movements in terms of classification accuracy. These results suggest that the BMI training will be useful for two important applications.

First, the non-invasive BMI training will be beneficial in training patients before applying invasive BMI. Previous studies demonstrated that the ability to control the BMI varies among patients (Yanagisawa et al., 2012a; Fukuma et al., 2016; Pandarinath et al., 2017). Before applying an invasive BMI for paralyzed patients, we need to evaluate their ability to control the BMI and to train them when the ability is poor. Our BMI training succeeded in improving the accuracy of controlling the BMI with improved cortical activities, which are also used for invasive BMI. Therefore, the proposed MEG-based BMI training will be beneficial for preoperative evaluation of the invasive BMI.

Second, the BMI training will be useful for inducing plastic changes in the cortical representation. Even for these subjects with normal motor function, the BMI training succeeded in improving the classification accuracy of the hand movements using the MEG signals. Our findings suggest that the BMI training did not induce the changes by normalizing the cortical activity but by modulating the activity depending on the decoder. The BMI training could be applied in clinical therapy to change maladapted cortical representation (Kuner and Flor, 2016).

Recent studies have revealed that BMI training in a closed-loop condition improves BMI performance. It has been demonstrated that closed-loop training improves the control of a neuroprosthetic device using multi-unit activities in accordance with some network plasticity and reorganization (Orsborn et al., 2014; Balasubramanian et al., 2017). Similarly, the performance of non-invasive BMI can be predicted by cortical activities and improved by closed-loop neurofeedback training (Hwang et al., 2009; Blankertz et al., 2010; Sugata et al., 2016; Wan et al., 2016). On the other hand, performance improvement depends on the properties of the cortical activities used by the BMI (Sadtler et al., 2014). Further studies are necessary to optimize the improvement of BMI performance for some clinical uses.

It should be noted that BMI training was effective to induce significant differences even with a limited number of subjects. Although the correct rate of robotic control varied among subjects, our BMI training induced a consistent effect on the correct rates. Indeed, our results successfully demonstrated that BMI training significantly improved classification accuracy during the offline task and the correct rates during the online BMI training even among a limited number of subjects.

In summary, neurofeedback training using MEG-based BMI provides a novel method to directly change the information content of motor representations by induced plasticity in the sensorimotor cortex.

## Data availability

The data that support the findings of this study are available on request from the corresponding author.

## Author contributions

RF and TaY performed the research, wrote the manuscript, and prepared all the figures. TaY designed the study. HY, MH, ToY, YS, YK, and HK reviewed the manuscript.

### Conflict of interest statement

The authors declare that the research was conducted in the absence of any commercial or financial relationships that could be construed as a potential conflict of interest.
